# The role of increased post-impact ball speed on plantar pressure during open and square stance groundstrokes in female tennis players

**DOI:** 10.1186/s13102-024-00919-0

**Published:** 2024-06-10

**Authors:** Johanna Lambrich, Thomas Muehlbauer

**Affiliations:** Division of Movement and Training Sciences, Biomechanics of Sport, University of Duisburg- Essen, Essen, Germany

**Keywords:** Racket sport, Stance style, Lower extremity, Pressure-detecting insoles, Plantar loading, Force, Biomechanics

## Abstract

**Background:**

It is firmly established that achieving a high ball speed during the execution of groundstrokes represents a relevant factor for success in tennis. However, little is known about how plantar pressure changes as post-impact ball speed is increased during open and square stance groundstrokes. The objective of the study was to determine how tennis players change the plantar pressure in each foot when they execute open versus square stance forehand groundstrokes in order to increase post-impact ball speed.

**Methods:**

Fifteen healthy female tennis players with ITN 2 or better (mean age: 22.7 ± 7.8 years) participated in this study. The players performed open and square stance longline forehand groundstrokes (topspin) at the following four post-impact ball speed levels: 80 km/h, 90 km/h, 100 km/h, and *v*_max_. Flexible pressure-detecting insoles were used to measure plantar pressure in each foot [i.e., dominant (equals the stroke arm) and nondominant].

**Results:**

The repeated measures ANOVA showed significant stance style × foot dominance interactions and post-hoc analyses revealed larger maximal and mean forces during open compared to square stance for the dominant but not non-dominant foot. Further, the ball speed × stance style × foot dominance interaction reached the level of significance and post-hoc analyses showed increased/decreased mean forces in the dominant/non-dominant foot during the square but not open stance when players increased their post-impact ball speed.

**Conclusion:**

Larger values in the open stance, but post-impact ball speed-adjusted values in square stance indicate different advantages in both styles, suggesting their situation-specific application.

## Background

When executing forehand groundstrokes in tennis, different stance styles are used, which can be divided into closed, neutral/square, semi open and open stance [1]. Although the open stance (i.e., hip parallel to the baseline) is predominantly used (60–70%) [1, 2], there is varying empirical support regarding biomechanical advantages compared to the square stance (i.e., hip perpendicular to the baseline), for example [3]. Specifically, Wang et al. [4] reported that the external rotation angular momentum of the shoulder joint was significantly larger with an open than a square stance. In contrast, Kawamoto et al. [5] found a significantly shorter duration from pelvis forward rotation to ball impact as well as a lower peak velocity of the torso’s centre of mass and the shoulder joint centre in the hitting direction for the open compared to the square stance. Lastly, Knudson and Bahamonde [6] detected no significant differences in racket velocity, vertical path of the racket, and trunk angular velocity at impact between the open versus square stance. These varying results may most likely be attributed to discrepancies in the used methodological approach such as players’ age (21–62 years), sex (i.e., male only), and performance level (i.e., intermediate, advanced, or professional) as well as the applied measurement devices (i.e., motion capture system or high-speed video recordings), analysed outcomes (e.g., measured or estimated values), and used stroke/ball velocities (e.g., fixed or maximal speed) [4–6].

In terms of stroke/ball velocity, there is evidence that leg kinetics change with increasing post-impact ball speed. Precisely, Lambrich and Muehlbauer [7] showed significantly increased (dominant foot) versus decreased (non-dominant foot) pressure values when female players (mean age: 21.7 ± 7.7 years) increased the post-impact ball speed from 100 km/h to *v*_max_ while performing the forehand groundstroke. Despite this gain in knowledge, unfortunately no specification was made regarding the used stance style. Instead, the players were free to decide their stance style (i.e., open, closed, or square).

Taking this deficit into account, the aim of the present study was to investigate how female tennis players change the plantar pressure in each foot when they execute open versus square stance forehand groundstrokes with the goal to increase post-impact ball speed. Based on a recent study [7], we hypothesised that plantar pressure values will increase in the dominant foot but decrease in the non-dominant foot when post-impact ball speed is increased, irrespective of stance style. On the basis of a previous study [8] stating that a forward movement for the square but an upward movement for the open stance is typical to generate stroke power, we further assumed that plantar pressure values will be higher in the latter one, regardless of post-impact ball speed.

The investigation of changes in plantar pressure during open and square stance longline groundstrokes while post-impact ball speed is increased is quite important from different perspectives. Specifically, for tactical reasons the use of different stance styles at variable stroke velocities is necessary for the success of tennis players [9]. From a theoretical perspective, the results of the present study can contribute to expand the existing biomechanical understanding of influencing factors on stroke performance. For instance, so far missing results of stance-specific kinetic analyses during progressively increased post-impact ball speed will be provided. From a practical perspective, information about speed-dependent differences between the open and square stance style can be used to develop stance-specific training exercises and to design exercise programs for an appropriate technique and fitness training.

## Methods

### Participants

Power analysis (G*Power, v3.1.9.7) showed that for a repeated measures analysis of variance (ANOVA) a minimum of 13 players would be required to detect significant differences (assuming Cohen’s *f* = 0.25, *α* err prob = 0.05, 1-*β* err prob = 0.80). The sample consisted of fifteen healthy female tennis players with an International Tennis Number (ITN) ≤ 2 competing in regular national tournaments. Characteristics of the participants are presented in Table 1. Twelve subjects were right-handed and three were left-handed. Participants’ written informed consent was obtained prior to the start of the study. The study was carried out according to the Declaration of Helsinki and the human ethics committee at the University of Duisburg-Essen, Faculty of Educational Sciences approved the study protocol.

**Table 1:** Characteristics of the study participants (*N* = 15)

Characteristic	Value
Age [years]	22.7 ± 7.8
Body height [cm]	171.6 ± 6.7
Body mass [kg]	65.6 ± 7.3
Training experience [years]	16.3 ± 7.2
Tennis training volume [hours/week]	10.3 ± 5.1
Athletic training volume [hours/week]	4.5 ± 3.4
International Tennis Number	≤ 2

Values are expressed as mean ± standard deviation

### Testing procedure

**Fig. 1:**
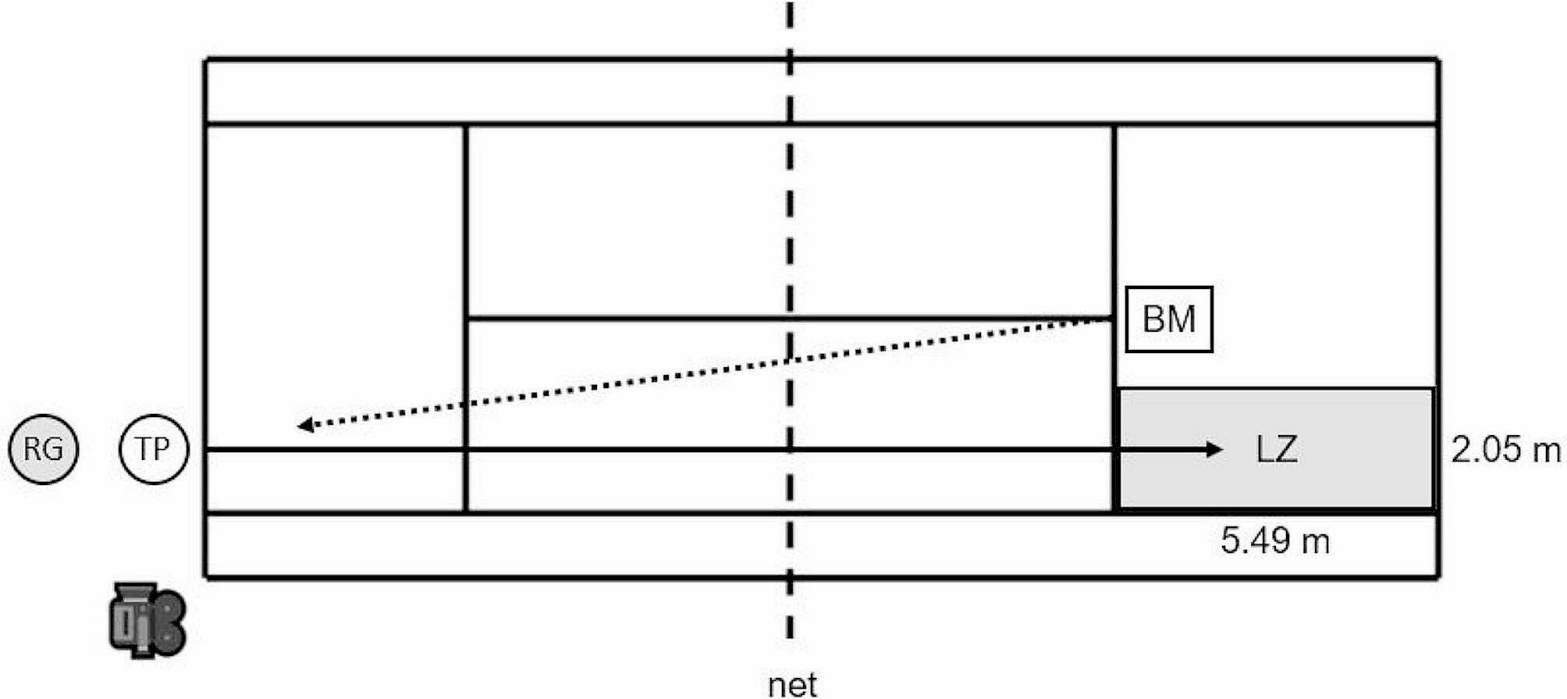
Experimental setup illustrating a tennis player performing a longline forehand groundstroke (topspin) using four post-impact ball speed levels (i.e., 80 km/h, 90 km/h, 100 km/h, *v*_max_). BM = ball machine; LZ = landing zone; RG = radar gun; TP = tennis player

The measurements were carried out on an indoor hardcourt. Each player used their own racket to ensure optimal stroke performance. The 5-min familiarisation phase included forehand topspin groundstrokes using the open and square stance. As described in one of our previous studies [7] the feed was standardised using a ball machine (Slinger Bag, Slinger, Windsor Mill, MD, USA) (speed: 40 km/h, feed: 15 balls/min). The testing of both stance styles (i.e., open and square) was conducted under the following four post-impact ball speed conditions: (a) 80 km/h, (b) 90 km/h, (c) 100 km/h, and (d) *v*_max_. A range of ± 2 km/h was allowed for the specified speed conditions a) to c). The order of the speed conditions was standardised, while the order of the stance styles was randomised. Stroke velocity was quantified to the nearest of 0.16 km/h using a “Stalker Pro” radar gun (Applied Concepts Inc., Richardson, TX, USA). The radar gun was positioned behind the player at a height of 1.8 m. New tennis balls were utilized for each player. Verbal feedback about ball speed was provided to the participants after each executed stroke. A valid trial included ten successful strokes per speed condition and stance style into a predefined 2.05 m x 5.49 m landing zone (Fig. 1). Subjects were given 60 s rest after each speed level and 120 s rest between stance styles.

### Assessment and analysis of plantar pressure data

Flexible instrumented insoles (GP MobilData WiFi, GeBioM mbH, Münster, Germany) with a sampling frequency of 200 Hz were utilized to quantify plantar pressure distribution. The collected data were transmitted to a laptop via wireless signal. The pressure-detecting insoles were positioned above the sole of the individual tennis shoes, which were designed for hard courts. Each subject wore the most fitting insole (e.g., the insole with length of 265 mm equals shoe size of EU 41–42). Synchronously, a video camera (iPad, Apple Inc., Cupertino, CA, USA) recorded the players’ stroke execution to detect the beginning and point of impact of the stroke movement. As in our previous work [7], this data was used for further analysis and interpolated to 0–100% of the stroke cycle. The data for the whole foot was analysed using MATLAB software version R2022b (The MathWorks Inc., Natick, MA, USA). For the dominant foot (equals the stroke arm) and the non-dominant foot, the force data were normalised to body weight and the following parameters were computed: maximum force (N/kg), mean force (N/kg), and force-time integral (Ns/kg). Maximum force identifies peak pressure points, which can highlight moments of high stress potentially linked to injury risks. Mean force provides an overall measure of the pressure exerted on the foot throughout the stroke, reflecting the general load experienced by the foot. The force-time integral combines both force and duration, offering a comprehensive view of the total load over time, which is essential for understanding the cumulative impact on the foot during repeated actions.Validity as well as reliability of the pressure-detecting insole system has been shown in a previous study [10].

### Statistical analysis

Descriptive statistics (i.e., mean and standard deviation) were computed using JASP version 0.16.4.0 (Amsterdam, The Netherlands), which was used for all analyses. For all analyses, assumptions of normality (Shapiro–Wilk Test) and homogeneity of variance/sphericity (Mauchly Test) were met prior to the application of inference statistics. Precisely, a 4 (ball speed: 80 km/h, 90 km/h, 100 km/h, *v*_max_) × 2 (stance style: open, square) × 2 (foot dominance: dominant, non-dominant) repeated measures ANOVA was performed. If a significant interaction occurred, Bonferroni-adjusted post-hoc analyses were executed. Further, GLM contrasts (type: simple) were analysed to investigate changes in plantar pressure outcomes with increased post-impact ball speed from 80 km/h (means the reference category) to 90 km/h, 100 km/h, and *v*_max_. For the ANOVA, the effect size partial eta-squared (*η*_p_^2^) was computed and classified as small (0.02 ≤ *η*_p_^2^ ≤ 0.12), medium (0.13 ≤ *η*_p_^2^ ≤ 0.25), or large (*η*_p_^2^ ≥ 0.26). For the post-hoc analyses, the effect size Cohen’s *d* was determined and interpreted as trivial (0 ≤ *d* ≤ 0.19), small (0.20 ≤ *d* ≤ 0.49), moderate (0.50 ≤ *d* ≤ 0.79), or large (*d* ≥ 0.80). The significance level was a priori set at *p* < .05 for all analyses.

## Results

**Fig. 2:**
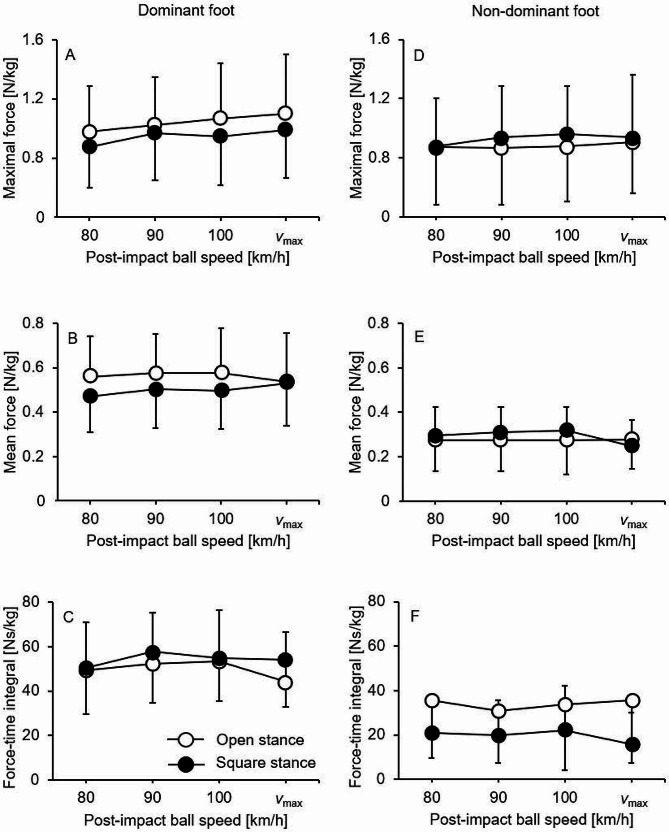
Plantar pressure values (mean and standard deviation) per post-impact ball speed level for the open (white circles) versus square (black circles) stance style by foot dominance (i.e., dominant vs. non-dominant) during the execution of longline forehand groundstrokes (topspin) in tennis

Descriptive (mean values ± standard deviations) and inference (repeated measures ANOVA) statistics are shown in Tables 2 and 3, respectively. The maximum post-impact ball speeds averaged at 132.8 ± 7.2 km/h (range: 118–148 km/h) and 137.1 ± 9.3 km/h (range: 120–160 km/h) for the open and square stance longline forehand groundstrokes (topspin), respectively. The changes in plantar pressure outcomes with increased post-impact ball speed during the open versus square stance longline forehand groundstrokes are displayed in Fig. 2A–F.

**Table 2:** Descriptive statistics of the plantar pressure data by post-impact ball speed level (i.e., 80 km/h, 90 km/h, 100 km/h, *v*_max_) and foot dominance (i.e., dominant vs. non-dominant) during open and square stance longline forehand groundstrokes (topspin) in tennis

Outcome	80 km/h			90 km/h				100 km/h			v_max_	
		Open	Square		Open	Square	Open		Square	Open		Square
*Dominant foot*												
Maximal force [N/kg]		0.98 ± 0.31	0.88 ± 0.28		1.02 ± 0.33	0.97 ± 0.32	1.07 ± 0.37		0.95 ± 0.33	1.10 ± 0.40		0.99 ± 0.33
Mean force [N/kg]		0.56 ± 0.18	0.47 ± 0.16		0.57 ± 0.18	0.50 ± 0.18	0.58 ± 0.20		0.50 ± 0.17	0.54 ± 0.22		0.53 ± 0.19
Force-time integral [Ns/kg]		49.41 ± 21.57	50.36 ± 20.81		52.46 ± 22.93	57.75 ± 23.15	53.55 ± 22.77		54.90 ± 19.32	44.19 ± 22.42		54.19 ± 21.18
*Non-dominant foot*												
Maximal force [N/kg]		0.87 ± 0.39	0.88 ± 0.32		0.87 ± 0.39	0.94 ± 0.35	0.88 ± 0.37		0.96 ± 0.33	0.91 ± 0.35		0.94 ± 0.42
Mean force [N/kg]		0.28 ± 0.14	0.29 ± 0.13		0.27 ± 0.14	0.31 ± 0.11	0.27 ± 0.15		0.32 ± 0.11	0.28 ± 0.13		0.25 ± 0.12
Force-time integral [Ns/kg]		35.67 ± 26.30	21.03 ± 14.87		30.70 ± 23.24	19.78 ± 15.69	33.67 ± 29.66		22.16 ± 20.09	35.40 ± 27.88		15.70 ± 14.28

Values are expressed as mean ± standard deviation

**Table 3:** Inference statistics for the main and interaction effects

Outcome	Main effect: BS	Main effect: SS	Main effect: FD	Interaction effect BS × SS	Interaction effect BS × FD	Interaction effect SS × FD	Interaction effect BS × SS × FD
Maximal force [N/kg]	< 0.001 (0.38)	0.457 (0.04)	0.066 (0.22)	0.647 (0.04)	0.314 (0.08)	0.032 (0.29)	0.805 (0.02)
Mean force [N/kg]	0.020 (0.21)	0.049 (0.25)	< 0.001 (0.89)	0.557 (0.05)	0.534 (0.05)	0.020 (0.33)	0.041 (0.18)
Force-time integral [Ns/kg]	0.013 (0.23)	0.025 (0.31)	< 0.001 (0.72)	0.320 (0.08)	0.635 (0.04)	0.065 (0.22)	0.472 (0.06)

Values are expressed as *p*-value (*η*_p_^2^-value). BS = ball speed; FD = foot dominance; SS = stance style

### Maximal force

There was a significant main effect of ball speed (*p* < .001, *η*_p_^2^ = 0.38) as well as a significant stance style × foot dominance interaction (*p* = .032, *η*_p_^2^ = 0.29). Post-hoc tests revealed significantly larger values during open compared to square stance for the dominant (80 km/h: *p* = .046, *d* = 0.34; 100 km/h: *p* = .027, *d* = 0.34; *v*_max_: *p* = .043, *d* = 0.28) but not for the non-dominant foot.

### Mean force

There were significant main effects of ball speed (*p* = .020, *η*_p_^2^ = 0.21), stance style (*p* = .049, *η*_p_^2^ = 0.25), and foot dominance (*p* < .001, *η*_p_^2^ = 0.89). Further, the stance style × foot dominance (*p* = .020, *η*_p_^2^ = 0.33) and the ball speed × stance style × foot dominance (*p* = .041, *η*_p_^2^ = 0.18) interactions reached the level of significance. Post-hoc tests revealed significantly larger values during open compared to square stance for the dominant (80 km/h: *p* = .006, *d* = 0.51; 90 km/h: *p* = .006, *d* = 0.41; 100 km/h: *p* = .001, *d* = 0.42) but not for the non-dominant foot. Moreover, mean force significantly changed (*p* = .018, *η*_p_^2^ = 0.56) during square but not open stance in the dominant foot when players increased their post-impact ball speed. GLM contrasts revealed significant increases from 80 km/h to 90 km/h (*p* = .022, *η*_p_^2^ = 0.32), 100 km/h (*p* = .046, *η*_p_^2^ = 0.26), and *v*_max_ (*p* = .002, *η*_p_^2^ = 0.50). For the non-dominant foot, mean force again significantly changed (*p* < .001, *d* = 0.79) during square but not open stance when post-impact ball speed was increased. However, GLM contrasts showed only a tendency toward a significant decrease from 80 km/h to *v*_max_ (*p* = .063, *η*_p_^2^ = 0.23).

### Force-time integral

There were significant main effects of ball speed (*p* = .013, *η*_p_^2^ = 0.23), stance style (*p* = .025, *η*_p_^2^ = 0.31), and foot dominance (*p* < .001, *η*_p_^2^ = 0.72) but no significant interaction effects.

## Discussion

To the authors’ knowledge, this is the first study that investigated changes in plantar pressure values as post-impact ball speed is increased (80 km/h, 90 km/h, 100 km/h, and *v*_max_) during open and square stance longline forehand groundstrokes (topspin) in elite female tennis players. The main findings of this study were (1) that during the square but not open stance, the mean forces increased in the dominant foot (equals the stroke arm) but decreased in the non-dominant foot when players increased their post-impact ball speed; (2) that in most speed conditions, maximal and mean forces were significantly larger during open compared to square stance for the dominant but not non-dominant foot.

Our first hypothesis stating that irrespective of stance style, the plantar pressure values will increase in the dominant foot but decrease in the non-dominant foot when post-impact ball speed is increased, was only supported with respect to the square stance. This result is contrary to one of our previous studies [7] which showed an increase in plantar pressure data for the dominant foot but a decrease for the non-dominant foot when post-impact ball increased from 100 km/h to *v*_max_, regardless of stance style. One possible reason could be that in square stance a large part of the force is gained by leg drive and weight shifting in stroke direction [5, 8]. In contrast, in the open stance, the force is generated by a more upward movement of the body [11]. In this regard, Kawatomo et al. [5] investigated 13 advanced male tennis players (mean age: 25.0 ± 2.5 years) and detected that the lack of weight shifting towards the hitting direction in the open stance was compensated for by the upward and sideways work of the torso.

The fact that the mean forces increased (dominant foot) / decreased (non-dominant foot) with increasing post-impact ball speed for the square stance, but remained unchanged for the open stance, indicates the potential for plantar pressure adjustments to changing ball speeds for the square stance. An application from this key finding is that a high post-impact ball speed can be primarily responded to by adapting pressure distribution between the legs. Accordingly, players who prefer to use the square stance should perform physical exercises to train different patterns of force generation for the dominant versus non-dominant leg.

In accordance with our second hypothesis, we detected larger values (i.e., maximal and mean forces) during open compared to square stance in most ball speed conditions. Although there are already two studies [12, 13] using kinetic analyses for different stance styles, there was no comparison of open versus square stance, but of attacking neutral, attacking open, and defensive open stance. Consequently, the aforementioned result can only be interpreted in the context of general statements that were made in handbooks on the biomechanics of tennis. In this regard, Diana Knudson [8] stated: *“A good leg drive forward (square stance) or more upward (open stance) is an important source of stroke power.”* (p. 93). Since only the vertical force component can be recorded using plantar pressure insoles, the previously formulated difference with regard to the movement direction is thus apparent in the force data. Significantly larger values for the open compared to the square stance were found only for the dominant but not for the non-dominant leg. This indicates different functions, with the dominant leg generating force and the non-dominant leg stabilizing the body [14]. An application from this finding could be that players who prefer to use the open stance style should perform physical exercises to differentially use both legs. For example, dynamic exercises (e.g., skipping, hopping, jumping) for the dominant leg and static exercises (balance, isometric strength) for the non-dominant leg [15].

Higher values were found for the maximum and mean force in the open compared to the square stance, but not for the force-time integral. This means that the general load and the peak load are greater in the open than the square stance. According to Martin et al. [12, 13] and Ellenbecker [16], these higher loads can result in an increased injury risk to the knee and hip in the open stance, particularly for the dominant foot. Therefore, tennis players who already suffer from knee or hip problems should be aware of this aspect and try to preferably use the square stance.

The present study has some limitations. First, our sample size (*N* = 15) was relatively small, although larger than in other studies [5, 6, 17] on this topic. Second, only female tennis players were examined, that show a larger mean quadriceps angle compared to men [18], indicating that the present findings cannot be transferred to male players. Third, plantar pressure data were collected from elite players (ITN ≤ 2), thus the results cannot be generalized to lower skill levels. Fourth, we restricted our assessment to biomechanical data concerning kinetics. Thus, future studies should use a combined approach including, kinetic, kinematic, and electromyographic data.

## Conclusions

In summary, this study investigated differences in plantar pressure values between the open and square stance while post-impact ball speed was systematically increased (80 km/h, 90 km/h, 100 km/h, and *v*_max_). We observed increased (dominant foot) and decreased (non-dominant foot) mean forces for the square but not open stance when female tennis players increased their post-impact ball speed. Further, we detected in most speed conditions significantly larger maximal and mean forces during open compared to square stance for the dominant but not non-dominant foot. Larger (open stance) and post-impact ball speed-adjusted (square stance) values indicate different advantages per stance style. Therefore, their application should be situation-specific.

## Data Availability

The data generated and analysed during the present study are not publicly available due to ethical restrictions but are available from the corresponding author upon reasonable request.

## Abbreviations

BM: Ball machine
D: Dominant foot
ITN: International Tennis Number
LZ: Landing zone
ND: Non-dominant foot
RG: Radar gun
TP: Tennis player
